# Genome-wide identification and characterization of Glutathione S-Transferases (GSTs) and their expression profile under abiotic stresses in tobacco (*Nicotiana tabacum* L.)

**DOI:** 10.1186/s12864-023-09450-x

**Published:** 2023-06-21

**Authors:** Zejun Mo, Ying Huang, Tianxiunan Pu, Lili Duan, Kai Pi, Jiajun Luo, Benshan Long, Anbin Lu, Renxiang Liu

**Affiliations:** 1 College of Tobacco, Guizhou University, Guiyang, China; 2 Key Laboratory of Tobacco Quality in Guizhou Province, Guiyang, China; 3 College of Agriculture, Guizhou University, Guiyang, China

**Keywords:** *Nicotiana tabacum* L., GST family, Characterization, Expression pattern, Abiotic stress

## Abstract

**Background:**

Glutathione S-transferases (GSTs) are large and multifunctional proteases that play an important role in detoxification, protection against biotic and abiotic stresses, and secondary metabolite transportation which is essential for plant growth and development. However, there is limited research on the identification and function of *NtGST*s.

**Results:**

This study uses K326 and other six tobacco varieties (Hongda, HG, GDH11, Va116, VG, and GDH88) as materials to conduct comprehensive genome-wide identification and functional characterization of the *GST* gene in tobacco. A total of 59 *NtGST*s were identified and classified into seven subfamilies via the whole-genome sequence analysis, with the Tau type serving as the major subfamily. The *NtGST*s in the same branch of the evolutionary tree had similar exon/intron structure and motif constitution. There were more than 42 collinear blocks between tobacco and pepper, tomato, and potato, indicating high homology conservation between them. Twelve segmental duplicated gene pairs and one tandem duplication may have had a substantial impact on the evolution and expansion of the tobacco *GST* gene family. The RT-qPCR results showed that the expression patterns of *NtGST*s varied significantly among tissues, varieties, and multiple abiotic stresses, suggesting that *NtGST* genes may widely respond to various abiotic stresses and hormones in tobacco, including *NtGSTF4*, *NtGSTL1*, *NtGSTZ1*, and *NtGSTU40*.

**Conclusions:**

This study provides a comprehensive analysis of the *NtGST* gene family, including structures and functions. Many *NtGSTs* play a critical regulatory role in tobacco growth and development, and responses to abiotic stresses. These findings offer novel and valuable insights for understanding the biological function of *NtGSTs* and the reference materials for cultivating highly resistant varieties and enhancing the yield and quality of crops.

**Supplementary Information:**

The online version contains supplementary material available at 10.1186/s12864-023-09450-x.

## Background

Plant secondary metabolites are a class of non-essential small-molecule organic compounds produced by secondary metabolic processes. Despite not being directly involved in the growth and development of plants, they are crucial for ecological adaptation [1]. Studies have shown that plant glutathione S-transferases (GSTs) are crucial for secondary metabolism and responding to various biotic and abiotic stresses [2, 3]. In both eukaryotes and prokaryotes, GSTs (EC2.5.1.18) are a superfamily of dimeric proteases with multiple biological functions [3, 4]. Up to 90 genes in plants encode GSTs, which is mainly distributed in the cytoplasm, with a few in the chloroplasts and the nucleus [5], and plays an important role in plant detoxification [6]. It catalyzes the covalent binding of reduced glutathione (GSH) with hydrophobic and electrophilic substrates to form conjugates that are isolated in vacuoles or transferred to the apoplast, thereby degrading both endogenous and exogenous harmful substances[2]. Simultaneously, in many plants, GSTs serve as a non-enzymatic catalytic carrier, allowing anthocyanins to combine with GSH and selectively transfer anthocyanins to vacuoles by ABC transporters [7–9].

Each subunit of glutathione transferase contains two N-terminal and C-terminal domains with different spatial structures [10]. The N-terminal consists of a β-sheet and an α-helix and is more conserved, whereas the latter consists of 4–7 α-helices, connected to the N-terminal domain by a short sequence of about ten amino acids [11]. The major differences in the structure and sequence of different *GST* genes are reflected in the N-terminal and C-terminal domains, which determine the substrate specificity [12]. In higher plants, based on the sequence characteristics of *GST* genes, they can be classified into eight typical subfamilies, including Tau (U), Phi (F), Lambda (L), Zeta (Z), Theta (T), elongation factor 1 gamma (EF1Bγ), dehydroascorbate reductase (DHAR), and tetrachlorohydroquinone dehalogenase (TCHQD) [13, 14]. However, in some species, *GST*s can be classified into 14 categories, where the remaining six include metaxin, Ure2p, microsomal prostaglandin E synthase type 2 (PGES2), hemerythrin, iota, and glutamate hydroxyquinone reductases (GHR) [15, 16]. The discovery and characterization of *GST* gene families have become increasingly precise in recent years due to species genome sequencing. Additionally, there are significant differences in the number and classification of *GST* genes among different species. According to existing reports, there are 79 *GST*s in rice [17], 55 *GST*s in Arabidopsis [18], 40 in radish [19], 22 in Salix [20], 73 in Medicago [21], and 38 in apple [22].

The ability of plants to adapt to adversity can be improved by the *GST* genes. The function of the *GST* gene family has been isolated and identified in many species, including Arabidopsis [23], cotton [24], Chinese cabbage [25], etc. According to studies, *GST*s enhance plant resistance to salt stress by elevating antioxidant enzyme activity, increasing chlorophyll content, reducing reactive oxygen species accumulation [26], and decreasing cell membrane damage [27]. Plant *GST* genes are crucial for maintaining the balance between cell turgor and reactive oxygen species [28], reducing oxidative damage caused by drought, and enhancing plant tolerance to drought stress [29, 30]. In response to low-temperature stress, *GST*s can catalyze the reaction of GSH with membrane lipid peroxides, reducing the damage that low-temperature stress causes to the cell membrane structure [7, 31]. The *GST* genes also involving the pathogen resistance [32], and it has found that the interaction between *TaGSTU6* and *TaCBSX3* may positively enhance wheat resistance to powdery mildew [33]. Furthermore, *GST*s are crucial in heavy metal stress [34, 35], herbicide detoxification [36], high temperature [37], light [38], and hormonal response [39].

Tobacco is a model plant for studying various biological phenomena and is an important leaf cash crop [40, 41]. For analyzing the expression pattern of the tobacco *GST* genes, many studies have focused on the cloning and validation of a single gene [42, 43]. However, there is no relevant report on the identification, characterization, and expression pattern analysis of the tobacco *GST* gene family. In this study, a comprehensive identification and analysis of the tobacco *GST* gene family have been conducted, including subfamily classification, gene naming, gene structure and motif, distribution on chromosomes, sequence comparison, phylogenetic tree, and the expression profile of *NtGST*s under different stresses have been analyzed. This can provide evidence for analyzing the role of the *NtGST*s and cultivating novel resistant tobacco varieties. At the same time, it is crucial to predict the function of the *GST* genes in other crops and improve the quality traits and stress resistance of crops.

## Results

### Identification and phylogenetic analysis of members of the *NtGST*s gene family

In this study, 59 *NtGST*s in seven subfamilies were identified in the genome of tobacco K326 and were named according to their location and order on chromosomes. Simultaneously, the physical and chemical properties and basic characteristics of these 59 genes were analyzed. Table S1 provides basic information on the length of the amino acid, isoelectric point (pI), protein molecular weight (Mw), and predicted subcellular location (Supplementary file 1). These NtGST proteins varied substantially in their amino acid composition, ranging from 61 to 512. Among them, *NtGSTZ2* was the smallest protein, encoding 61 amino acids, and *NtGSTL1* was the largest protein, encoding 512 amino acids. Mw varied significantly among different genes, with the mean Mw of 28264.41 KDa, the lowest Mw of 6909.98 KDa (*NtGSTU39*), and the highest Mw of 59313.6 KDa (*NtGSTU1*). The isoelectric point (pI) varied significantly among the same subfamilies, ranging from 4.63 (*NtGSTL3*) to 9.44 (*NtGSTL2*). Subcellular localization predictions found that there were 43 NtGST proteins located in the cytoplasm, with six *NtGST* located in the chloroplast, six in the nucleus, two (*NtGSTL2* and *NtGSTZ3*) in the chloroplast thylakoid membrane, one (*NtDHAR2*) in the mitochondrion, and one (*NtGSTZ4*) in the organelle membrane.

**Fig. 1:**
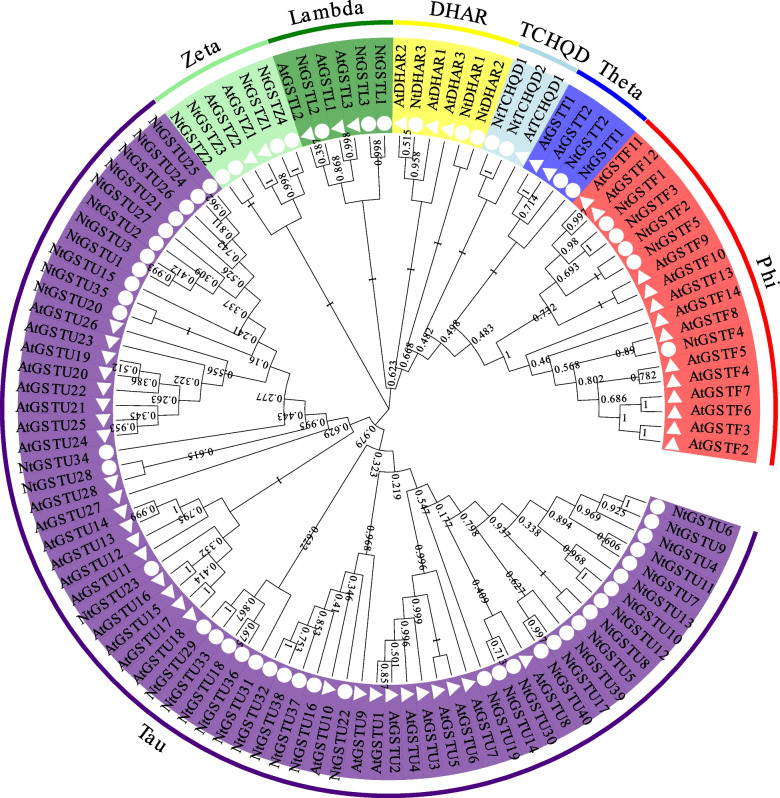
Unrooted phylogenetic tree representing the relationships among 59 *NtGST*s in tobacco and 56 *AtGST*s in Arabidopsis. The genes in tobacco are marked with a dot, while those in Arabidopsis are marked with a triangle. The different color blocks represent different subfamilies

To investigate the phylogenetic relationship of tobacco GST proteins, we constructed an evolutionary tree of 59 tobacco and 56 Arabidopsis GST proteins using the maximum likelihood method (Fig. 1). All identified *NtGST*s were divided into seven subfamilies, including Tau subfamily (U), Zeta subfamily (Z), Lambda subfamily (L), Theta subfamily (T), Dehydroascorbate reductase subfamily (DHAR), Tetrachlorohydroquinone dehalogenase-like subfamily (TCHQD), and Phi subfamily (F). Among them, the Tau subfamily was the largest group, representing 67.8% of the number of all NtGST proteins (*NtGSTU1–40*). Moreover, five proteins (*NtGSTF1–5*) were classified as Phi subfamily, three proteins (*NtGSTL1–3*) as Lambda subfamily, four proteins (*NtGSTZ1–4*) as Zeta subfamily, two proteins (*NtGSTT1–2*) as Lambda subfamily, three proteins (*NtDHAR1–3*) as DHAR subfamily, and two proteins (*NtTCHQD1–2*) as TCHQD subfamily. The phylogenetic relationship showed that the tobacco NtGST proteins had high homology with Arabidopsis AtGST proteins, suggesting that the evolution of *GST* genes between the two species was conservative and may have the same biological functions.

### Gene structure and motif composition of the NtGST proteins

**Fig. 2:**
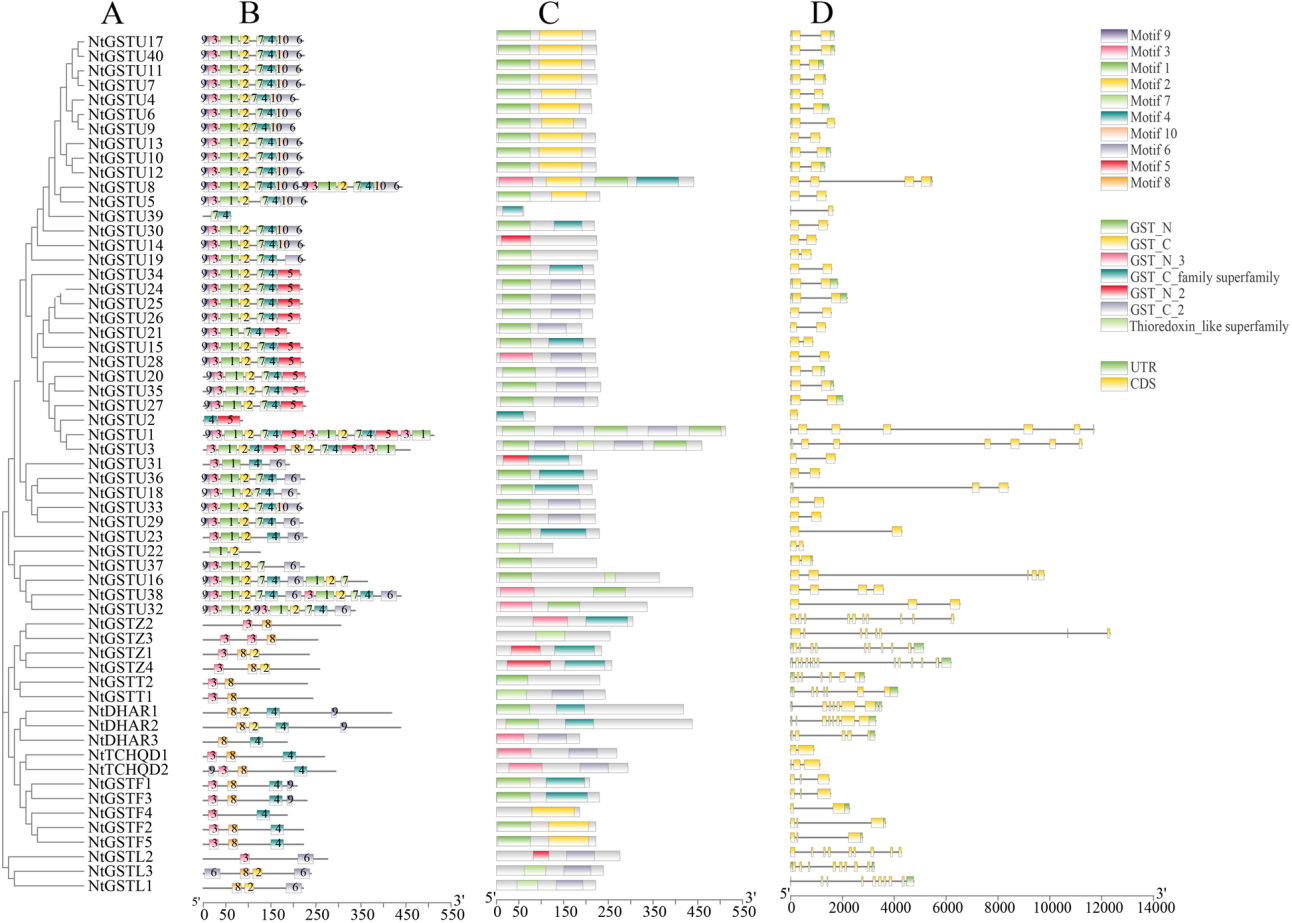
Phylogenetic, gene structure, and motif analyses of the *NtGST* genes. (**A**) The phylogenetic analysis of *NtGST* family genes in tobacco. (**B**) Amino acid motifs in the *NtGST* proteins (1–10) are represented by coloured boxes. Black lines indicate relative protein lengths. (**C**) Conserved domains of the *NtGST* genes. (**D**) Exons and introns are indicated by yellow rectangles, and black lines, respectively

A total of 10 conserved motifs were identified in the *NtGST*s and named motif 1 to motif 10 to better understand tobacco NtGST proteins’ function. The findings suggested that those genes in the same branch of the phylogenetic tree contained similar motifs, suggesting that these genes might perform similar biological functions. Except for a few genes, the remaining *NtGST*s contained conserved motif 3. Most genes also contained motif 2, suggesting that these motifs can be used as markers to recognize *NtGST*s (Fig. 2A, 2B). Among the tobacco NtGST proteins, the Tau subfamily covers most motif types. The motifs annotation results showed that motifs 1, 3, and 5 were annotated as the GST-N domain, and motifs 2, 4, and 6 were annotated as the GST-C domain. After the conserved domain prediction and analysis of 59 tobacco NtGST proteins, all proteins were found to contain both GST-N and GST-C domains (Fig. 2C).

The genomic sequences of tobacco NtGST proteins were examined to determine the number and structural information of their introns and exons. The findings revealed that all the NtGST proteins contained introns except for the *NtGSTU2*, and the numbers ranged from 1 to 12. About 57.6% of the *NtGST*s contained only one intron (Fig. 2D, Table S1). Furthermore, the number and structure of introns/exons with similar evolutionary degrees were also similar. There were many introns and exons in the Zeta subfamily, Lambda subfamily, and DHAR subfamily, among which the Zeta subfamily had the largest number, with an average of 10 exons and nine introns, and *NtGSTZ4* had 12 introns. In the Tau subfamily, most tobacco NtGST proteins, except for some genes, contained two exons and one intron. Furthermore, 32 NtGST proteins (54.2%) had untranslated region (UTR).

### Distribution of chromosomes and duplication events analysis of the NtGST proteins

**Fig. 3:**
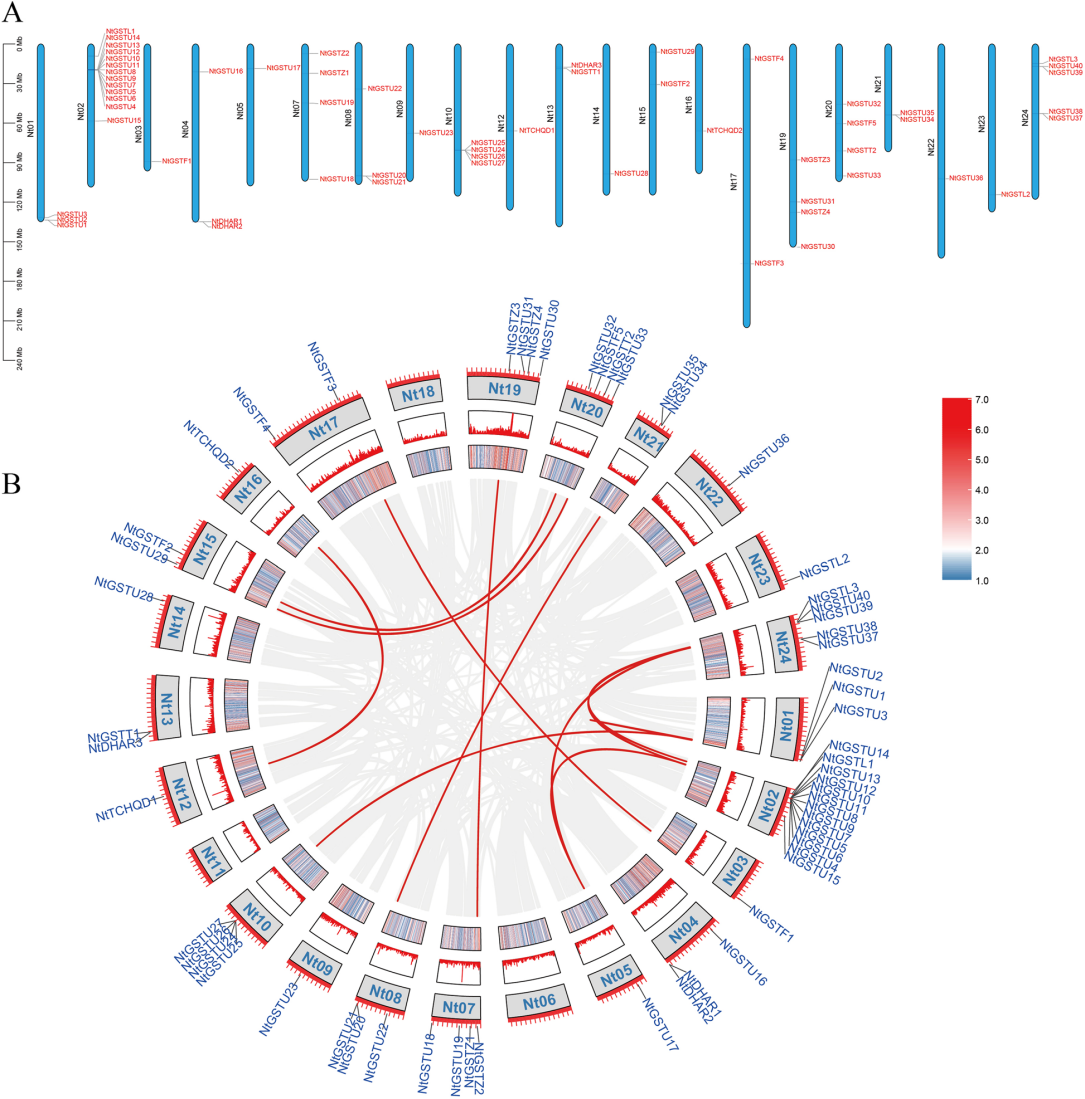
Distribution on chromosomes and duplication events analysis. **A** Schematic representations of the chromosomal distribution of the *NtGST*s*.* The blue vertical bars represent the tobacco chromosomes, Chromosome number is indicated to the left of each chromosome (black), and gene number is indicated to the right of each chromosome (red). **B** Schematic representations of the interchromosomal relationships of the *NtGST*s. The grey lines represent all colinear blocks in the tobacco genome, and red lines represent duplicated GST gene pairs

Based on the genomic information of tobacco K326, chromosome localization analysis of tobacco *GST* protein was performed to further understand its distribution on the chromosome. The findings suggested that 59 NtGST proteins were localized on 21 chromosomes, and the number of proteins on each chromosome was significantly different (Fig. 3A). Most of the NtGST proteins (13, 22.0%) were distributed on chromosome 2, followed by distribution on chromosome 24 (5 NtGST proteins). There were four NtGST proteins distributed on chromosomes 7, 10, 19, and 20, respectively, three NtGST proteins on chromosomes 1, 4, and 8, and two NtGST proteins on chromosomes 13, 15, 17, and 21. Moreover, one NtGST protein was distributed on chromosomes 3, 5, 9, 12, 14, 16, 22, and 23, respectively. However, no protein was distributed on chromosomes 6, 11, and 18. During the evolution of species, gene duplication or loss might lead to the absence of NtGST proteins on some chromosomes. Furthermore, it was found that the distribution of NtGST proteins on chromosomes was not directly associated with chromosome length, size, etc.

Segmental duplication often occurs during chromosome rearrangement, resulting in a large number of duplicated chromosome blocks in the genome. However, multiple members of adjacent homologous gene families amassed on one chromosome results in tandem duplication [44]. Tandem and segmental gene duplication can generate a large number of gene families and promote their amplification, which is critical for species genome evolution [45]. The duplication events of NtGST proteins at the genome-wide level were investigated to reveal the amplification mechanism of the tobacco *GST* gene family. The results revealed that twelve pairs of segmental duplicated genes and one pair of tandem duplication were unevenly distributed on 21 chromosomes and were connected by red curves (Fig. 3B; Supplementary file 2, Table S2), suggesting that the amplification of the tobacco *GST* gene family was associated with gene duplication events, and the segment duplication was the primary driving force for the generation of new members of the *NtGST* gene family.

### Collinearity analysis of the NtGST proteins in tobacco

**Fig. 4:**
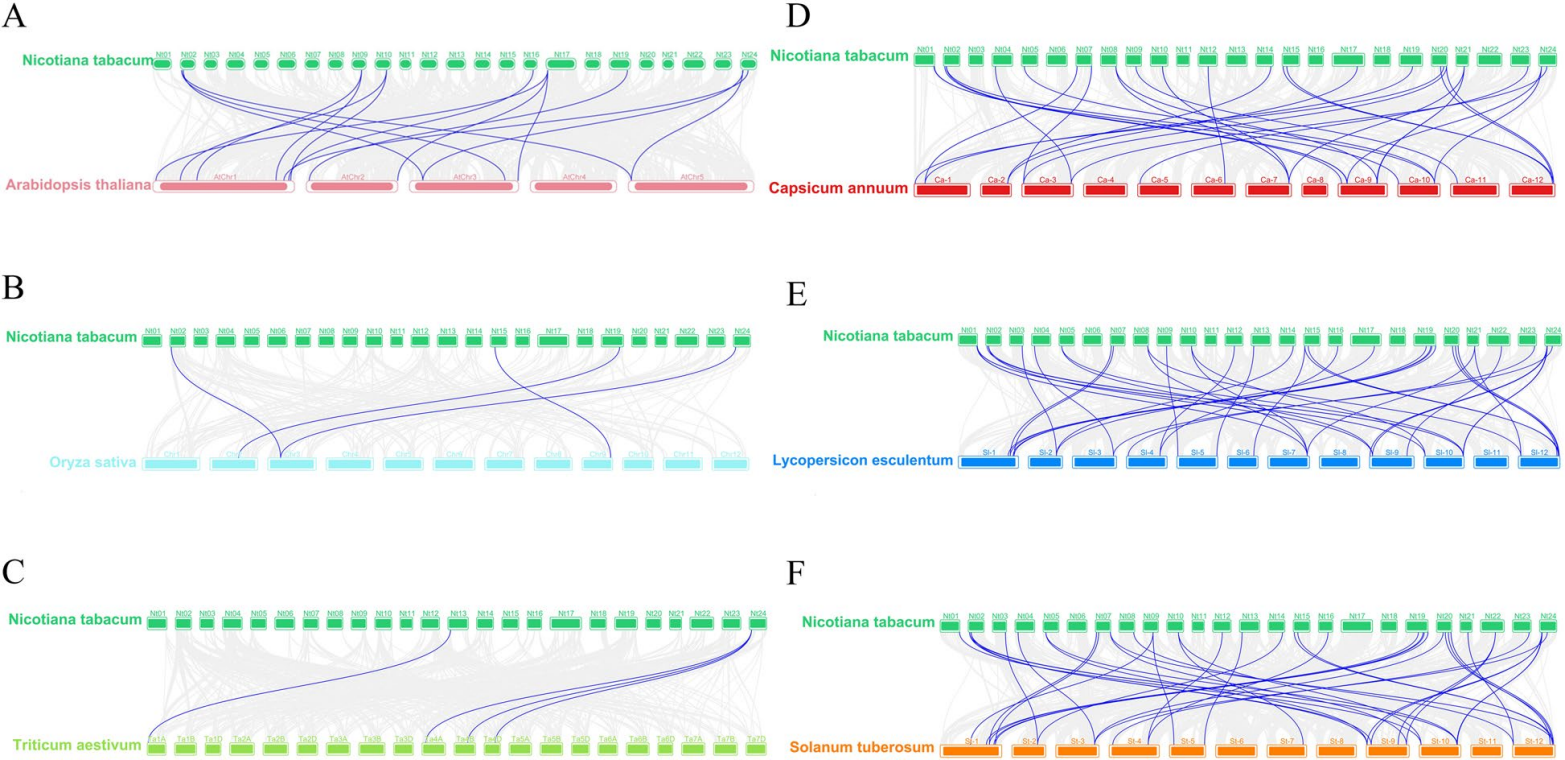
Collinearity analysis of GST genes between *Nicotiana tabacum* (tobacco) and six other species. **A**, **B**, **C**, **D**, **E**, and **F** are collinearity comparisons with *Arabidopsis thaliana* (arabidopsis), *Oryza sativa* (rice), *Triticum aestivum* (wheat), *Capsicum annuum* (pepper), *Lycopersicon esculentum* (tomato), and *Solanum tuberosum* (potato), respectively. The gray lines in the background represent the collinearity block in the genome of tobacco and other plants, while the blue lines highlight the collinearity GST gene pairs

According to the evolutionary relationship and motif composition of GST proteins in different plants, one dicotyledonous plant (*Arabidopsis thaliana*), two monocotyledonous plants (*Oryza sativa* and *Triticum aestivum*), and three Solanaceae plants (*Capsicum annuum*, *Lycopersicon esculentum*, and *Solanum tuberosum*) were selected for collinearity analysis to investigate the collinearity relationship between *GST* gene members and *NtGST*s in different species and their evolutionary origins. The findings revealed that 47 *NtGST*s displayed a collinearity relationship across the six species, indicating that during the process of evolution, tobacco *GST*s were highly conserved on the corresponding chromosomes. A total of 15, 4, 4, 42, 47, and 49 collinear blocks of *NtGST* genes were identified in *Arabidopsis thaliana*, *Oryza sativa*, *Triticum aestivum*, *Capsicum annuum*, *Lycopersicon esculentum*, and *Solanum tuberosum*, respectively (Supplementary file 3, Table S3). Based on these collinearity results, six collinear maps were drawn, representing homologous gene pairs in tobacco and other species connected by blue lines (Fig. 4). The results revealed that there were only a few collinear gene pairs between tobacco and monocotyledonous plants (*Oryza sativa* and *Triticum aestivum*), indicating that most *GST* genes were formed after the differentiation of dicotyledonous and monocotyledonous plants. However, there were more than 42 collinear blocks between tobacco and *Capsicum annuum*, *Lycopersicon esculentum*, and *Solanum tuberosum*, showing a high homology of the *GST* genes at the same family level.

### Prediction of *cis*-acting element analysis of tobacco NtGST proteins

**Fig. 5:**
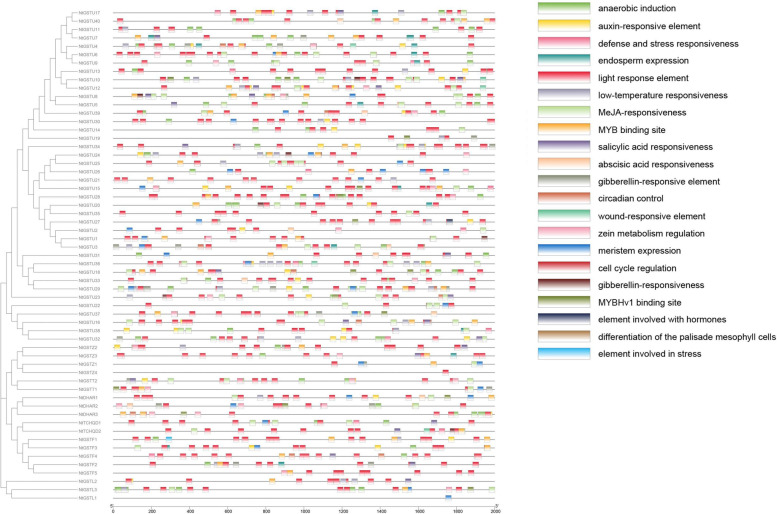
The *cis-*acting element analysis of 2000-bp sequences upstream of *NtGST* genes

The *cis*-acting elements include promoters, enhancers, regulatory sequences, and inducible elements, which primarily regulate target gene expression. The promoter sequence 2000 bp upstream of the transcription start site was selected and the *cis*-regulatory elements of 59 NtGST proteins were analyzed using the plant CARE website. The prediction revealed that these *cis-* acting elements primarily included hormone-related response elements such as auxin, abscisic acid (ABA), salicylic acid (SA), methyl jasmonate (MeJA), and gibberellin acid (GA), stress-related response elements such as light, dehydration, low-temp, and salt stresses, and wound-responsive elements (Fig. 5, Supplementary file 4, Table S4). Furthermore, these *cis-*acting elements were also found to contain elements related to the cell cycle, meristem expression, endosperm development, and circadian response. These findings indicated that *NtGST*s could be crucial for response to hormones, stress conditions, plant growth and development, and wound induction.

### Transcriptome expression profile of NtGST proteins in different materials and tissues

**Fig. 6:**
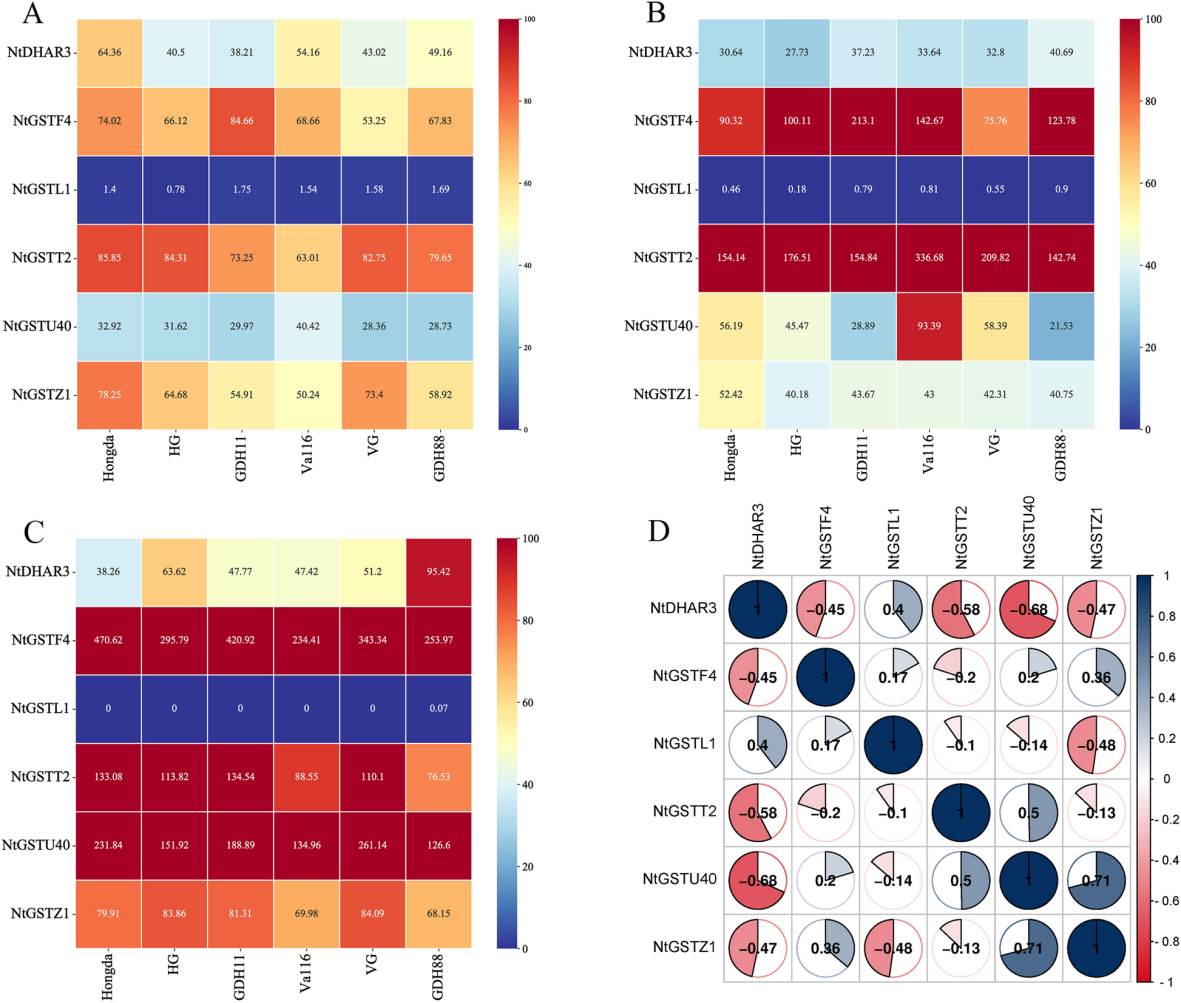
Transcriptome expression profile of *NtGST* genes in different materials and tissues. The data is expressed in FPKM values using transcriptome sequencing. The abscissa represents the material name (Hongda, HG, GDH11, Va116, VG, and GDH88, respectively). The ordinate represents gene name (*NtDHAR3*, *NtGSTF4*, *NtGSTL1*, *NtGSTT2*, *NtGSTU40*, and *NtGSTZ1*, respectively). The (**A**), (**B**), and (**C**) are the expression profiles of six *NtGST* genes in the leaves, stalks, and roots, respectively. (**D**) Positive number: positively correlated; negative number: negatively correlated

To further verify the function of tobacco GST protein, we selected 6 genes with relatively highest or lowest expression levels on different chromosomes and in different subfamilies from 59 *NtGST*s, and analyzed their transcriptome expression profiles in different tissues (roots, stalks, leaves) and different materials (Hongda, HG, GDH11, Va116, VG, and GDH88). The results showed that the expression patterns of six *NtGST*s in the same tissue were significantly different, indicating that *NtGST*s served different physiological functions in tobacco (Figs. 6A, B, and C). In other words, except *NtGSTL1*, the other five genes were expressed in three tissues, with significant expression in roots, followed by stems, and finally in leaves. *NtDHAR3* showed a high expression level in the root of GDH88. The expression pattern of *NtGSTZ1* in tobacco plants revealed that the root had higher expression than the leaf, with the stem having the lowest expression. *NtGSTF4*, *NtGSTT2*, and *NtGSTU40* had the highest expression levels in the three tissues, especially *NtGSTU40*, which had a certain tissue expression tendency to roots. At the same time, the expression pattern of the same gene in different materials was relatively consistent, indicating that these genes were highly conservative. Most *NtGST*s were negatively correlated in the three tissues; for example, the expression level of *NtDHAR3* was negatively correlated with *NtGSTZ1*, *NtGSTF4*, *NtGSTT2*, and *NtGSTU40* (Fig. 6D). Furthermore, some genes were positively correlated in the three tissues, such as *NtGSTU40*, which showed a positive correlation with *NtGSTZ1* and *NtGSTT2*.

### Expression pattern analysis of *NtGST* genes in response to temperature

**Fig. 7:**
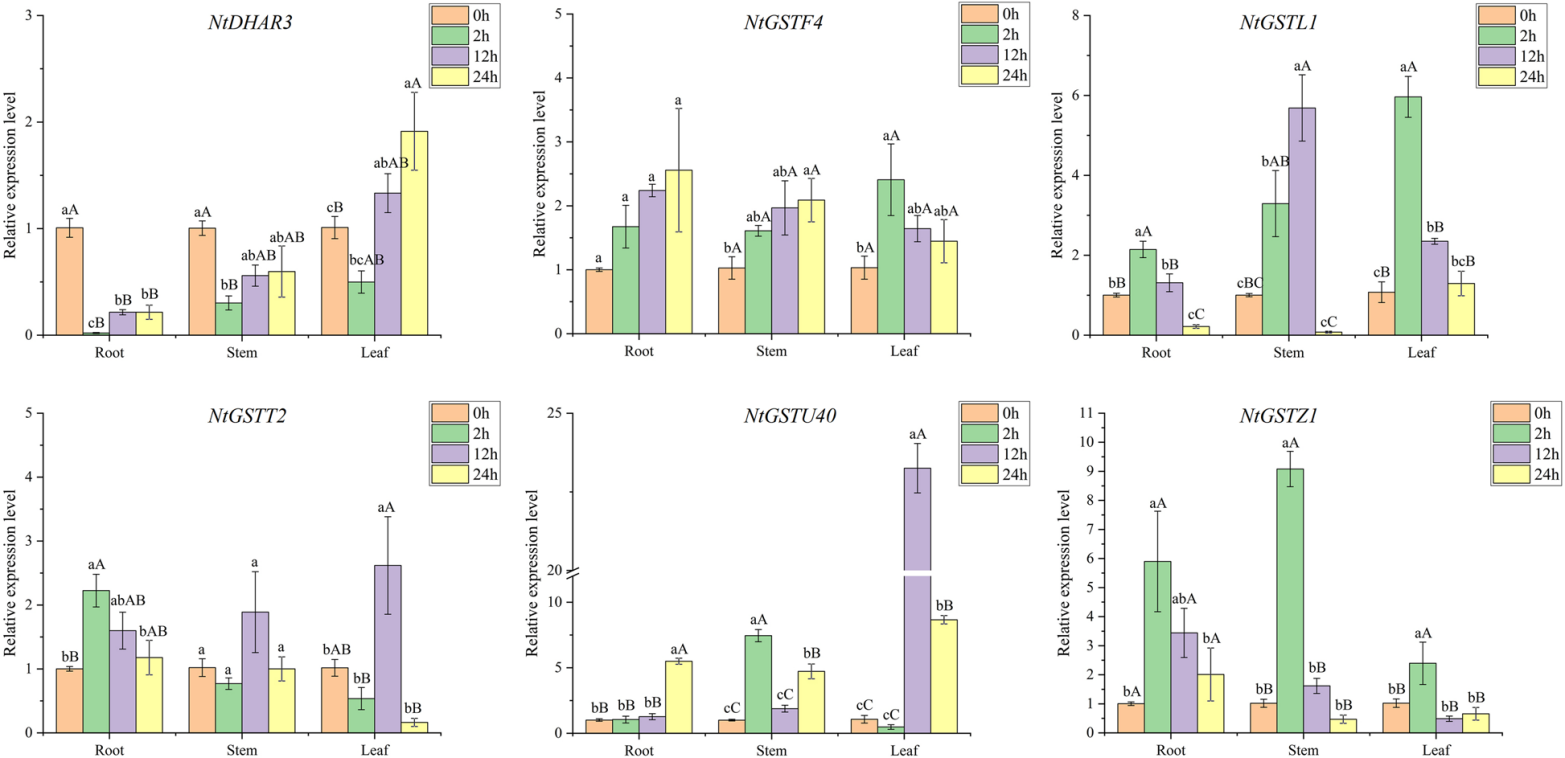
Gene expression pattern of six *NtGST* genes in response to low temperature (4 °C) in roots, stalks, and leaves. Error bars represent significant differences among the three replicates. Significant differences in the gene expression level at *P* < 0.05 and *P* < 0.01 were determined using Duncan’s new multiple-range tests. The lowercase alphabets represent a significant difference (*P* < 0.05), while the uppercase alphabets represent a highly significant difference (*P* < 0.01). Similarly hereinafter

**Fig. 8:**
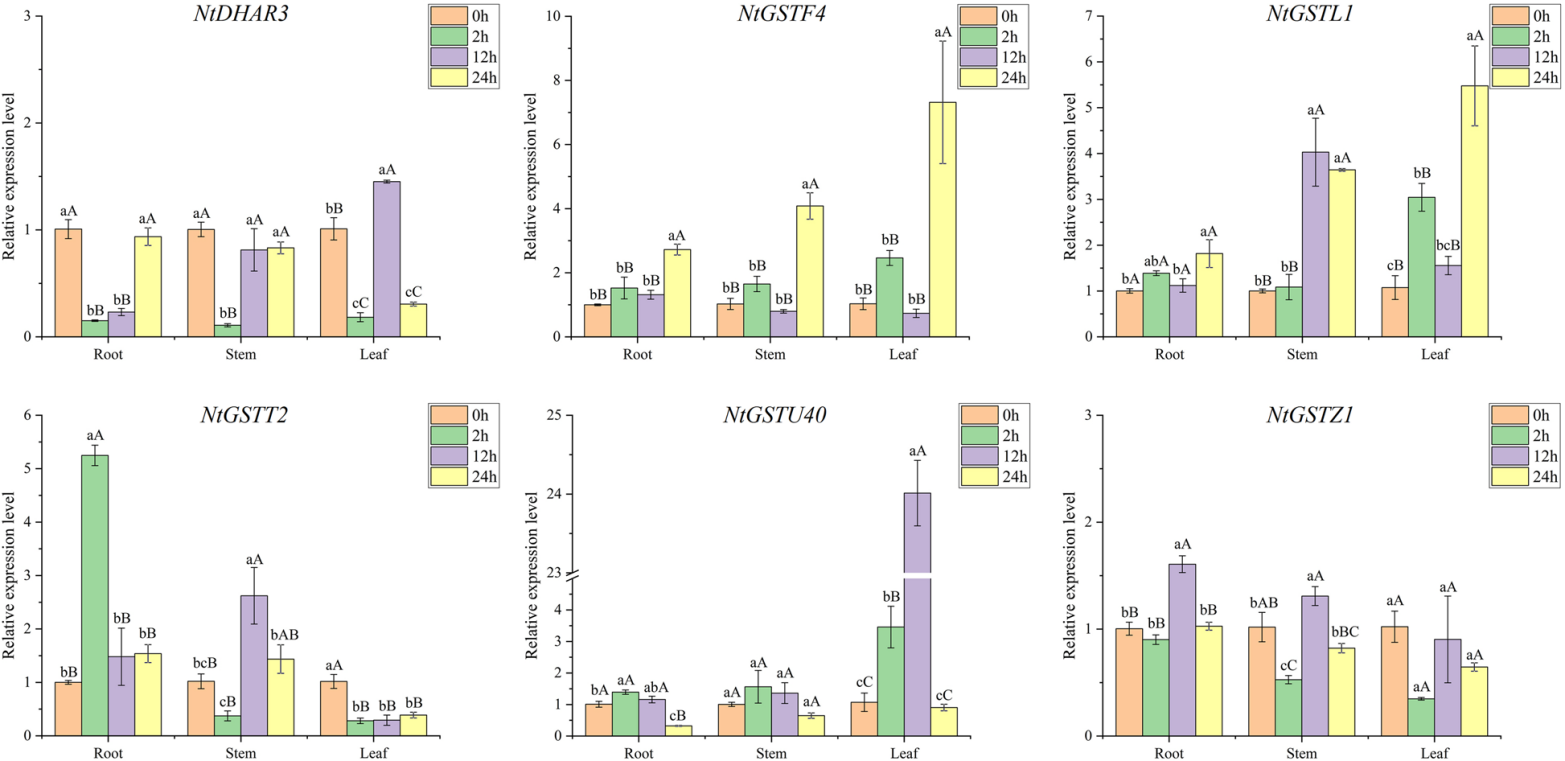
Gene expression pattern of six *NtGST* genes in response to high temperature (38 °C) in roots, stalks, and leaves

Previous *cis-*acting element prediction results showed that *NtGST*s had a certain response effect on temperature. To study the expression patterns of these genes in response to temperature stress, tobacco plants were treated with low (4 °C) and high temperatures (38 °C), and the expression level of six *NtGST*s was measured using qRT-PCR. The results revealed that different *NtGST*s expression patterns varied in high and low temperatures, as well as in roots, stalks, and leaves. Under low-temperature treatment, the expression pattern of *NtGSTL1*, *NtGSTT2*, and *NtGSTZ1* was relatively consistent, whether in the root, stalks, or leaf and indicated stimulated expression in the early stress response stage. Moreover, the expression level was first increased and then decreased (Fig. 7). While *NtGSTF4* expression was consistently induced in the root and stem, the expression level in the leaves was first increased and then decreased. *NtGSTU40* was highly expressed in the leaves after 12 h of treatment, more than 20 times higher than in the control group. Also, at high-temperature treatment, different *NtGST*s showed different response capacities (Fig. 8). In general, most genes, including *NtDHAR3*, *NtGSTF4*, *NtGSTL1*, and *NtGSTZ1*, responded significantly in the later stage of stress. However, the expression of *NtDHAR3* was inhibited in the early stage. In all three tissues, *NtGSTT2* and *NtGSTU40* were first increased and then decreased, and these two genes were induced in the roots and leaves at 12 h after treatment. These findings indicated that *NtGST*s have a certain response to temperature stress and could, under necessary conditions, enhance the plant’s defense against extreme temperature.

### Expression pattern analysis of *NtGST* genes in response to salt and drought

**Fig. 9:**
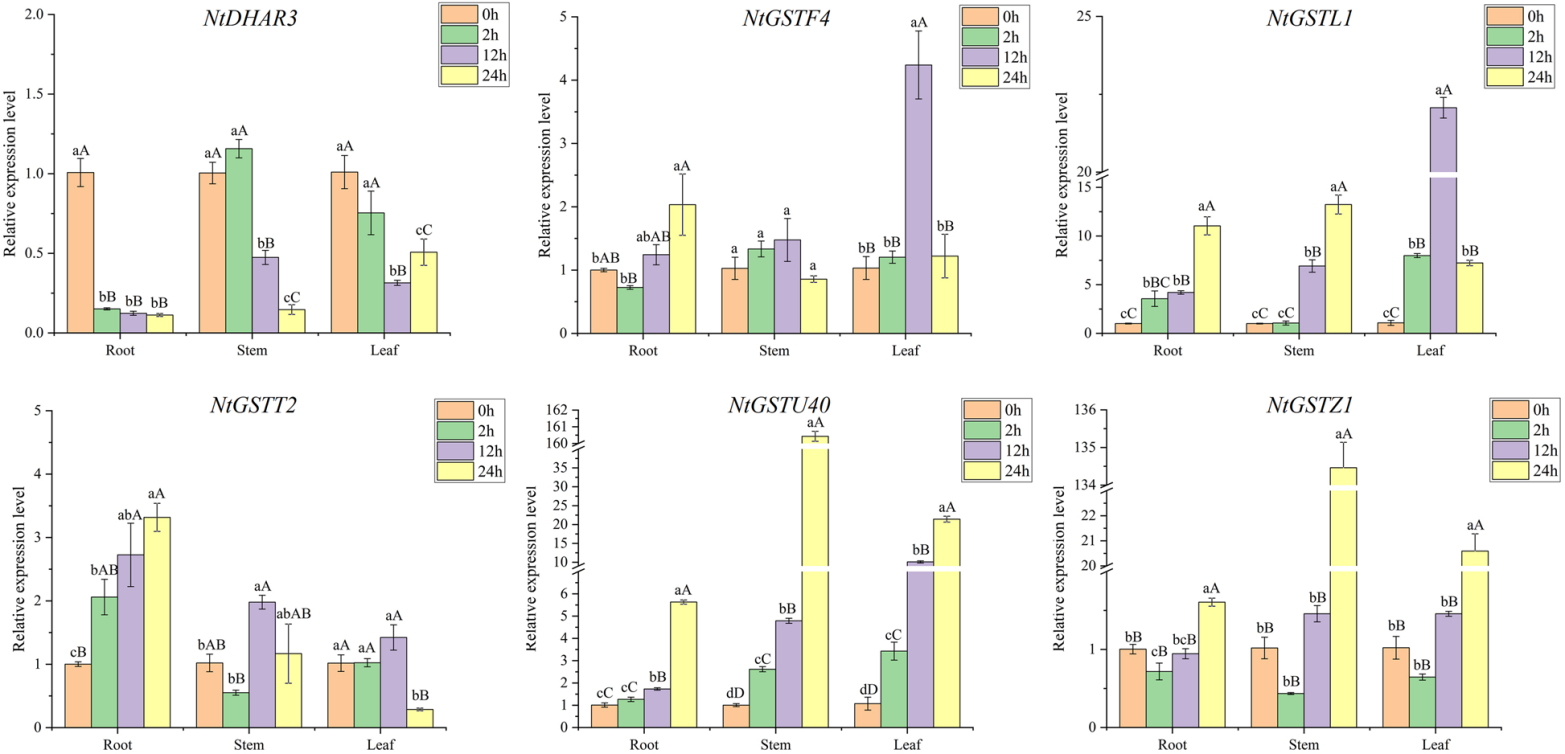
Gene expression pattern of six *NtGST* genes in response to salt in roots, stalks, and leaves

**Fig. 10:**
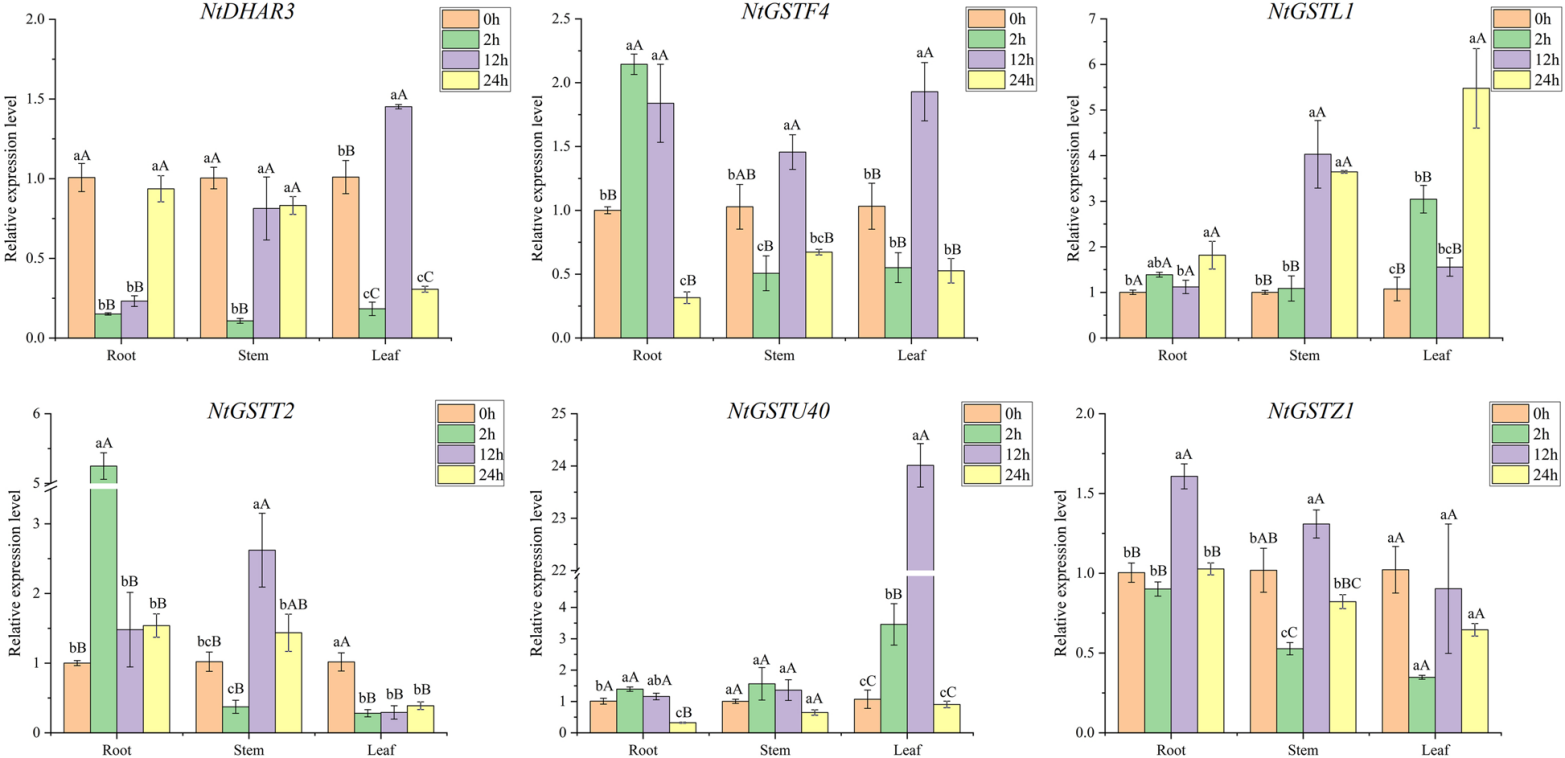
Gene expression pattern of six *NtGST* genes in response to drought in roots, stalks, and leaves

Drought is the primary environmental stress factor that restricts plant growth and causes crop yield reduction. In arid and semi-arid areas, drought generally occurs simultaneously with soil salinization, significantly impacting plants [46]. To study the response of *NtGST*s to drought and salt stress, the expression of six *NtGST*s was measured, and the expression pattern was analyzed. The results revealed that each gene had significant differences in response to salt and drought in different tissues, and the time of stress response varied as well. Under salt stress, the expression patterns of *NtGSTU40* and *NtGSTZ1* were relatively similar, being highly induced in all three tissues at 24 h after the treatment, with the highest expression in the stalk, more than 100 times that in the control group, followed by leaves, and then the root (Fig. 9). However, with the delay in treatment time, the expression level of *NtGSTU40* increased continuously, while *NtGSTZ1* decreased first and then increased. *NtGSTF4*, *NtGSTL1*, and *NtGSTT2* showed a trend of increasing first and then decreasing in leaves, with the highest expression at 12 h after the treatment. However, the expression level of *NtDHAR3* was inhibited in the roots, stems, and leaves. Under drought stress, most genes responded in the early, and middle stages of stress in the three tissues, including *NtGSTF4*, *NtGSTT2*, *NtGSTU40*, and *NtGSTZ1*, whose expression levels were increased first and then decreased (Fig. 10). *NtGSTU40* demonstrated a more than 20-fold increase in expression in leaves 12 h after treatment compared to the control group. However, *NtDHAR3* showed a trend of first decreasing and then increasing, and the maximum expression was only 1.4 times that of the control, which showed inhibition in the three tissues. In general, *NtGST*s have a certain response to salt and drought, and different genes have different response effects. These genes could enhance the defense ability of tobacco plants against salt and drought to some extent.

### Expression pattern analysis of *NtGST* genes in response to hormones

**Fig. 11:**
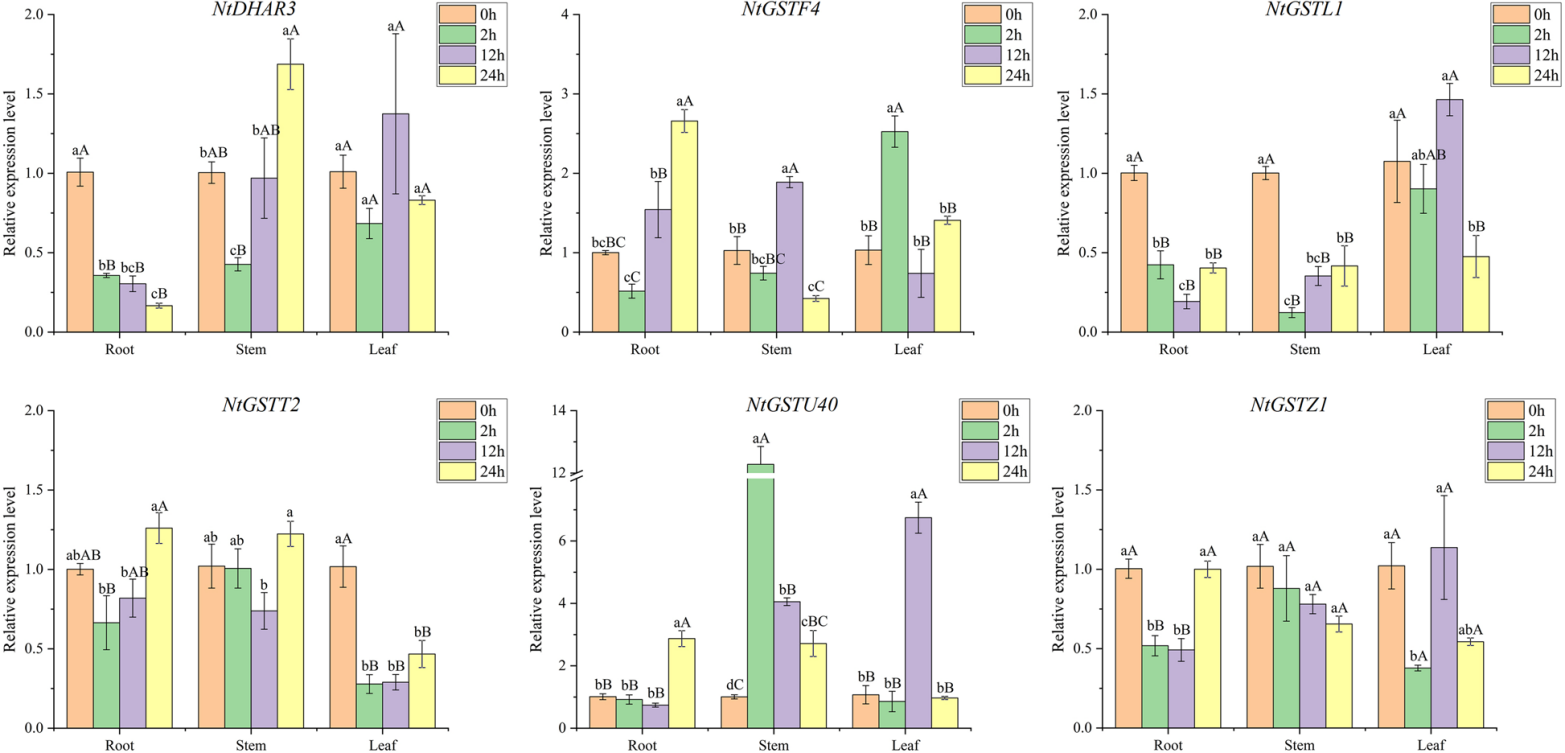
Gene expression pattern of six *NtGST* genes in response to IAA in roots, stalks and leaves

**Fig. 12:**
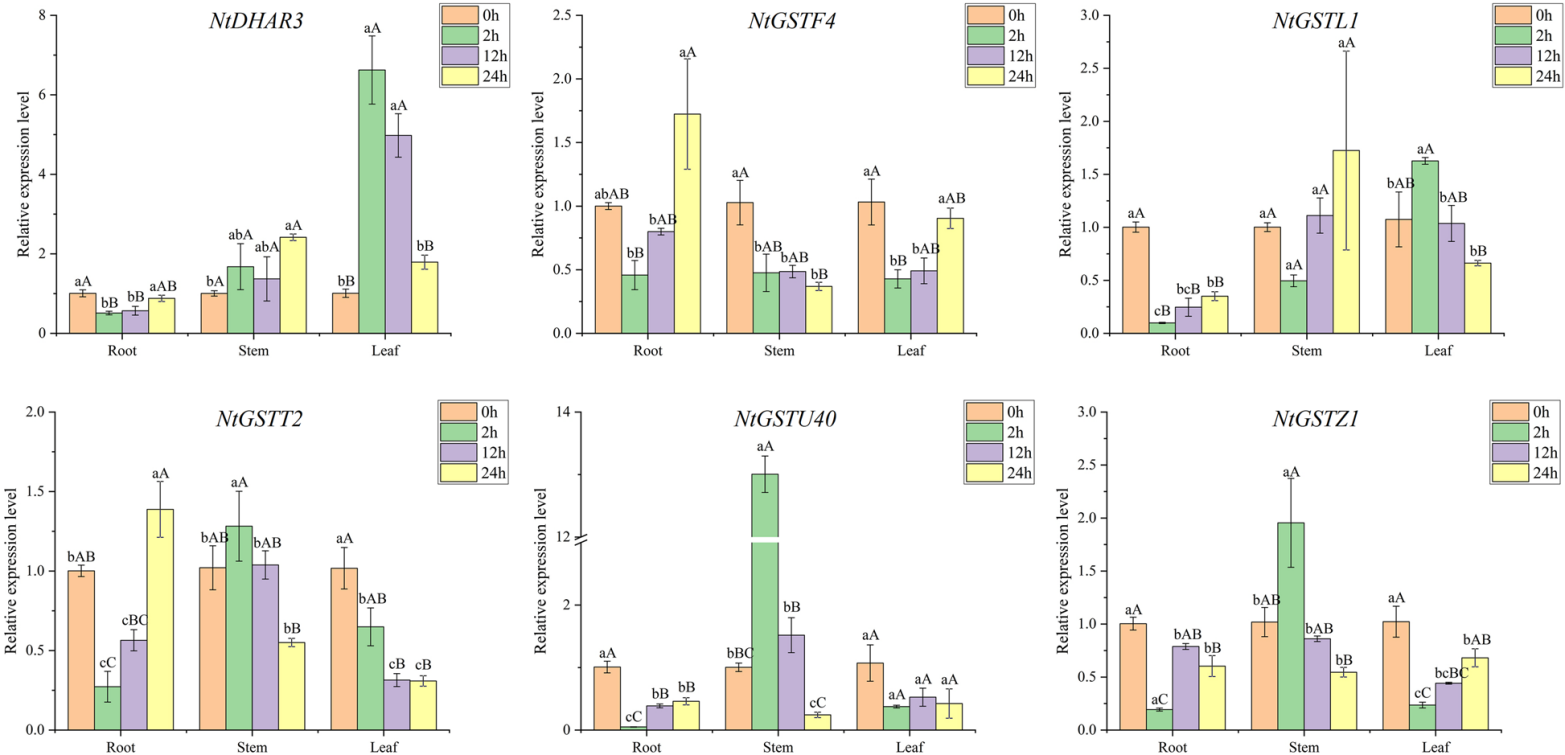
Gene expression pattern of six *NtGST* genes in response to SA in roots, stalks and leaves

Hormones can jointly participate and regulate plant growth and development and improve their adaptability to the environment. The promoter sequence of *NtGST*s was found to contain many hormone-related response elements, such as auxin, abscisic acid, salicylic acid, and methyl jasmonate (Fig. 5). To investigate the expression pattern of *NtGST*s after hormone treatment, tobacco seedlings were treated with indole-3-acetic acid (IAA) and salicylic acid. The results demonstrated that the expression of *NtGST*s was induced by hormones and SA, with significant induction by SA. Most genes responded to hormone stress mainly at 2 h and 12 h after treatment. Under IAA treatment, the induced expression of *NtGST*s included up-regulation and down-regulation, and the expression levels of different genes differed (Fig. 11). The maximum expression time of different genes in root, stem, and leaf tissues also differed; for example, *NtGSTF4* was expressed after 24 h, 12 h, and 2 h of treatment, respectively. Under SA stress, *NtDHAR3*, *NtGSTF4*, *NtGSTU40*, and *NtGSTZ1* expressions were significantly induced, and most of them showed a trend of first decreasing and then increasing (Fig. 12). *NtGST*s often respond to hormones differently, which reflects their ability to regulate plant growth and development.

## Discussion

Plant GSTs are a large, ancient and multifunctional gene family. In addition to being crucial for secondary metabolism and multi-drug detoxification, it is critical in responding to biotic and abiotic stresses [19, 47, 48]. The *GST* family genes have been identified and studied in various plants due to their special and abundant functions, and the number of *GST* identified in different plants varies greatly. However, the tobacco *GST* genes research is mostly focused on verifying the role of a single gene because its genetic transformation is easy. Therefore, we conducted systematic studies on the tobacco *GST* gene family.

Based on tobacco genome sequence and RNA seq data, 59 *NtGST*s were found. Comparing the number of *GST* genes in different plants, the evolution of *NtGST*s can be analyzed. Fifty-five *AtGST* genes were identified in *Arabidopsis thaliana* [18], 79 *OsGST*s in *Oryza sativa* [49], 90 *SlGST*s in *Lycopersicon esculentum* [48], 85 *CaGST*s in *Capsicum annuum* [37], 90 *StGST*s in *Solanum tuberosum* [50], and 346 *TaGST*s in *Triticum aestivum* [51]. The genome size of these plant species (*Nicotiana tabacum*, *Arabidopsis thaliana*, *Oryza sativa*, *Lycopersicon esculentum*, *Capsicum annuum*, *Solanum tuberosum*, and *Triticum aestivum*) was 4.5 Gb [52], 117 Mb [52], 466 Mb [53], 900 Mb [54], 3.5-Gb [55], 1.67 Gb [56], and 16 Gb [57], respectively. We speculated that there may be a relationship between the size of the species genome and the number of *GST* genes. For instance, Arabidopsis has the smallest genome and the fewest *GST*, whereas wheat has the largest genome and the most *GST* genes. In Arabidopsis, the *GST* genes were classified into seven subfamilies. However, compared with the plants belonging to the Solanaceae (potato, ten subfamilies; tomato, ten subfamilies; pepper, ten subfamilies), none of the genes belonged to the subfamilies of EF1Bɣ1, GHR, MGST, and others. This may be due to the loss of the *NtGST*s in the evolutionary process or differentiation into other subfamily members. A total of 40 *NtGST*s in tobacco come from Tau (U) subfamily, and Tau subfamily members are the most common in other plants, with the largest number, indicating that *NtGST*s conforms to the distribution characteristics of *GST* gene subfamilies in plants.

The 59 *NtGST*s were distributed on twenty-one chromosomes, and no *NtGST* was found on the other three chromosomes, suggesting an important role of of gene family expansion. During species evolution, gene duplication events can result in the expansion of a gene family, including tandem duplication, segmental duplication, transposition events, and genome-wide duplication [45, 56]. In most polyploid plants, segmental duplication occurs frequently [37]. In this study, twelve pairs of segmental duplicated genes and one pair of tandem duplication were found to be unevenly distributed on 21 chromosomes in the *NtGST* family. The findings indicated that these gene replication events were crucial in the evolution of the tobacco *GST* gene family, with the segment duplication event playing a leading role. We hypothesized that this might be because tobacco is allotetraploid, and the results of gene replication events are consistent with those in pepper and wheat [37, 58]. The *NtGST*s were primarily distributed in the U-type and F-type subfamilies. Hence it can also be speculated that the expansion of the *GST* family of tobacco is mainly due to the duplication events of these two families, which is also consistent with that of cotton [24]. Genes with replication events are often accompanied by the loss or addition of functions, the latter may be the result of the two genes showing different functions in the duplicated gene pair, and gene expression patterns often reflect their biological functions [54].

In this study, ten motifs in 59 *NtGST*s were found, and genes in the same branch of the evolutionary tree contained similar motif constitutions, indicating that they may perform similar functions in plants. According to gene function studies, *GST* family members can participate in various biotic and abiotic stresses, including mechanical damage, heavy metals, drought, temperature, and phytohormones [18, 37]. *Cis-*acting elements are crucial in regulating plant adaptation to stress, growth, and development by participating in the expression of target genes [59]. Through the analysis of upstream *cis-*acting elements, it was found that the members of the tobacco *GST* gene family contain various environmental stress response elements, including hormones, stress, MYB binding site, circadian rhythm, and injury induction. This result was consistent with the promoter analysis results of gene families such as *SlGST*s and *AtGST*s, indicating that these response elements directly determine the transcriptional expression level and response-ability of *GST* family genes under stress[48].

Analysis of gene expression patterns can reveal crucial information about the physiological function of genes. Most of the tobacco *GST* gene family members were found to be expressed normally in different cultivars, and only individual genes, such as *NtGSTU40* in the stack of Va116 and *NtDHAR3* in the root of GDH88, displayed specific expression. Simultaneously, we found that the expression of *NtGST*s have tissue specificity. Tobacco roots had a higher expression level than stalks, followed by leaves. These results were consistent with those of soybeans [60] and peppers [37], indicating that *NtGST*s are crucial in the growth and development of tobacco. This may be because the root is the key organ for the absorption and transportation of water and mineral nutrients in plants and also the most important organ for recognizing and feeling injuries, which can quickly respond to stress [61, 62]. Moreover, this result also reflects the possible functions of the *GST* gene family.

Several studies have shown that GSTs play multiple functions in many aspects of growth and development in plants. Yang et al. demonstrated that the *JrGSTU1* gene in walnut (*Juglans regia* L.) could enhance the *GST* activity to regulate the expression of other stress- related genes and thus reduce the levels of reactive oxygen species [7]. To evaluate the potential biological function of the *NtGST* genes and their ability to respond to stress, we examined the expression patterns of six *NtGST*s in response to six stresses. The results showed that the expression patterns of different genes varied under different stresses, which were consistent with previous studies[24, 50]. For example, *NtGSTF4* expression was up-regulated in the roots, stems, and leaves under cold stress, while *NtGSTL1* and *NtGSTZ1* were first increased and then decreased. Furthermore, the response times of the same gene to low and high temperatures differed. For example, *NtGSTZ1* responded to low temperature at 2 h after treatment, while its maximum response to high temperature was at 12 h after treatment. Interestingly, *NtGSTU40* exhibited the maximum expression level in the leaves under low- or high-temperature stresses, and the highest stress response occurred at 12 h after treatment. These results indicated that the *NtGST*s might be involved in a complex cross-regulatory network in plants.

Salt and drought, the two primary abiotic stresses, have serious impacts on plant growth and crop yield [46]. Under salt stress, many genes showed strong stress responses, including *NtGSTF4*, *NtGSTT2*, *NtGSTL1*, *NtGSTU40*, and *NtGSTZ14*, exhibiting an overall upward trend. Previous studies have shown that the heterologous expression of the lambda subfamily, *VvGSTF13* or *JrGSTT1*, in tobacco enhanced the tolerance to salinity and drought stresses[63–65]. This finding was consistent with our results. Although Moons et al. [66] claimed that genes in the DHAR subfamily were significantly up-regulated under drought stress, *NtDHAR3* showed a down-regulated expression pattern under salt and drought stresses, indicating that the expression pattern of *GST* family genes would also vary by species. Under the treatment of hormones (IAA and SA), *NtGSTU40* was significantly expressed, which was consistent with the expression of *TaGSTU62* and *OsGSTU4* in rice [67]. Therefore, we hypothesized that the *NtGSTs* might function similarly to other plant *GST* genes.

In this study, a comprehensive and systematic identification and analysis of the *NtGSTs* was conducted to provide a reference for studying the *GST* gene function in plants. The potential role of *NtGST*s in plant resistance to stress was verified using RT-qPCR analysis. However, the mechanism of action of *NtGST*s in response to stress requires further investigation for better application to the cultivation of highly resistant cultivars and thus improve crop yield and quality.

## Conclusions

In summary, a comprehensive characterization and analysis of the *NtGST*s at the genome level were performed in this study. A total of 59 *NtGST*s were identified and categorized into seven subfamilies, and information on physicochemical properties and subcellular localization for these genes was provided. The *NtGST*s in the same branch of the evolutionary tree have similar exon/intron structures and motif constitutions. And the 59 *NtGST*s were unevenly distributed on 21 chromosomes. The analysis of their collinearity with the *GST* gene in other plants provided an important reference for studying the evolutionary characteristics of *NtGST*s. Furthermore, twelve pairs of segmental duplicated genes and one pair of tandem duplication were unevenly distributed on 21 chromosomes, suggesting that gene duplication events are crucial in the evolution and expansion of the tobacco *GST* gene family. The expression of *NtGST*s in different tissues and varieties and the response to multiple stresses and hormone treatment indicated that *NtGST*s play a critical regulatory role in tobacco growth and development. These results provide novel and valuable insights for studying the biological function of *NtGST*s.

## Materials and methods

### Plant materials: planting, treating and sampling

In this experiment, seven tobacco varieties, including K326, Hongda, HG, GDH11, Va116, VG, and GDH88, were selected to determine the function of *NtGST*s. The K326 was used for different stress treatments, and the remaining six varieties were used to analyze the expression pattern of *NtGST*s in different tissues and stages. All materials were provided by Key Laboratory for Tobacco Quality Research Guizhou Province and cultivated in the plant culture laboratory. The collection of plant material, experimental research, and field studies on plants were complied with relevant institutional, national, and international guidelines and legislation. Using floating dish breeding, sowing and seedling raising were performed under environmental conditions, including 75% relative humidity, 16 h/28 °C during the day, and 8 h/20 °C at night. On the 75th day of transplantation, three consistently growing plants in each replicate were sampled from three tissues (roots, stalks, and leaves), and three biological replicates were set.

Based on the chromosome location information of 59 *NtGST*s, combined with the results of their transcriptome analysis in roots, stems and leaves, we focused on exploring the potential biological functions under abiotic stress of six genes with relatively highest or lowest expression levels in different subfamilies located on different chromosomes. When the tobacco plants grew to the sixth real leaf, the same growing plants were selected and treated with high temperature (38 °C), low temperature (4 °C), salt (5% NaCl), drought (15% PEG6000), and hormone (100 µmol/L IAA and SA). Three biological replicates were set for each treatment, and samples were taken at 0 h, 2 h, 12 h, and 24 h after treatment. The collected samples were immediately frozen in liquid nitrogen and stored in a hypothermia refrigerator at -80 °C for real-time fluorescence quantitative PCR (RT-qPCR) experiments.

### Identification of the *GST* genes of Tobacco

The genome sequence was obtained from the tobacco genome project website (https://solgenomics.net/organism/Nicotiana_tabacum/genome), and the hidden Markov model (HMM) profile of the GST domains (PF00043 and PF02798) was obtained from the Pfam protein family database (http://pfam.sanger.ac.uk/). The *NtGST*s were searched in the tobacco genome database using the HMMER3.0 software with the default parameters (the cutoff value of 0.01). Meanwhile, using the sequence information in PF00043 and PF02798 files as templates, BLASTP software was used to compare the entire tobacco genome and screen out proteins containing these two sequence information. The results of BLASTP and HMMER 3.0 were integrated and analyzed. Pfam and SMART programs were used to detect candidate genes that may contain GST domains obtained from the BLASTP and HMMER 3.0 analysis results. Each potential gene was then manually examined to ensure predicted conserved sequences in the N and C terminal regions of the GST domain. A total of 59 *NtGST*s were identified from the tobacco genome via testing and screening. The molecular weight (MW) and isoelectric points (pl) of the *NtGST* family members were predicted and analyzed using the online analysis software ExPASy (http:/www.expasy.org/tools/), and BUSCA (http://busca.biocomp.unibo.it/) was used for subcellular localization prediction.

### Phylogenetic analysis and gene structure

A multiple sequence alignment of the obtained GST protein sequences and the previously reported Arabidopsis thaliana sequences was performed using the ClustalW default parameters. Subsequently, the amino acid sequence of the deduced GST domain was adjusted using GeneDoc software. To determine the exon–intron structure of the *NtGST*s, the predicted coding sequence was compared with the corresponding full-length sequence using Gene Structure Display Server (GSDS: http://gsds.cbi.pku.edu.cn) [68]. The online software MEME (http://meme-suite.org/tools/meme) was used for the conserved domain analysis with the number of motifs set to 10 and the motif width range set to 50–100 amino acids [69].

### Chromosomal mapping, gene replication, and syntenic analysis with other plant species

Based on the tobacco annotation data obtained from the Solanaceae database, the data of starting position of the *GST* genes on the chromosome was obtained. The gene homologous duplication events were then obtained according to the alignment between the pairwise genes. Finally, the chromosomal mapping and alignment results were visually analyzed using online bioinformatics tools. A multiple collinear scanning tool, MCScanX, was used to analyze the gene duplication events [70]. The *GST* collinear analysis atlas was constructed using the Dual Systeny Plotter software, and the *GST* collinear relationship of tobacco with *Arabidopsis thaliana*, *Oryza sativa*, *Triticum aestivum*, *Capsicum annuum*, *Lycopersicon esculentum*, and *Solanum tuberosum* was determined, respectively.

### The *cis-*acting element analysis

A total of 2000 bp gene sequences upstream of the *NtGST*s were obtained from the Solanaceae database. These sequences with *cis-*acting elements were analyzed using the PlantCare online software and visualized using the bioinformatics software.

### Transcriptome sequencing analysis

The Hongda, HG, GDH11, Va116, VG, and GDH88 were used for transcriptome sequencing analysis. The transcriptome data used in this study is derived from the previous experimental data of our research group. The samples were root, stalks and leaf tissues, and the sampling stage was one week after topping. The sequencing of biological samples was entrusted to Shanghai Majorbio Company. Data analysis was conducted using the method of Mo et al. [71]. The original data has been uploaded to the NCBI public database. The names of the repository/repositories and accession number(s) can be found below: https://www.ncbi.nlm.nih.gov/geo/query/acc.cgi?acc = GSE233199.

### Expression of the *NtGST* genes according to real-time fluorescence quantitative PCR

Six genes were randomly selected for RT-qPCR experiments to determine the expression levels of *NtGST* genes under different abiotic stresses. The experiments were performed using the SYBR Premix Ex Taq kit (Takara) and the Applied Biosystems 7500 Real-Time PCR system (Life Technologies Corporation, Beverly, MA, USA). Primer Premier 6 was used to design the gene primers. The gene names and corresponding primers used for the qPCR test are listed in Supplementary file 5. The 2^−ΔΔCT^ method was used to determine the relative expression level of each gene [72].

### Statistical analysis

Duncan’s new multiple range test was conducted to analyze the variation in the gene expression levels (*P* < 0.05 and *P* < 0.01) using the SPSS (version 16.0) software. Origin 2018 (95_64) and Adobe Illustrator CS6 were used for figure drawing.

## Supplementary Information

**Additional file 1: Table S1.** List of the 59 *NtGST* genes identified in this study.

**Additional file 2: Table S2.** List of the tandem repeat gene pairs of *NtGSTs* in tobacco.

**Additional file 3.** The lists of Nicotiana tabacum and other species GST synteny gene pairs.

**Additional file 4: Table S4.** Analysis of *cis*-acting elements in promoters of GST gene family members.

**Additional file 5: Table S5.** Primer sequences of six selected genes used in real-time qPCR analysis.

## Data Availability

The whole *Nicotiana tabacum* genome sequence information was obtained from the solgenomics website (https://solgenomics.net/organism/Nicotiana_tabacum/genome) and the website is open to all researchers. The tobacco materials (K326, Hongda, HG, GDH11, Va116, VG, and GDH88) used in this study were supplied by Prof. Renxiang Liu of Guizhou University. The datasets supporting the conclusions of this article are included in the article and its Supplementary files.

## Abbreviations

BUSCA: Bologna unified subcellular component annotator
GSDS: Gene structure display server
GSH: Glutathione
GST: Glutathione S-transferases
HMM: Hidden markov model
HMMER: Biosequence analysis using profile hidden markov models
IAA: Indole-3-acetic acid
MEGA: Molecular evolutionary genetics analysis
MEME: Multiple expectation maximizations for motif elicitation
Mw: Protein molecular weight
MYB: V-myb avian myeloblastosis viral oncogene homolog
PEG: Polyethylene glycol
pI: Isoelectric point
RT-qPCR: Real-time quantitative PCR
SA: Salicylic acid
SMART: Simple modular architecture research tool
SPSS: Statistical product and service solutions
UTR: Untranslated region

## References

[1] Khare S, Singh NB, Singh A, Hussain I, Niharika K, Yadav V (2020). Plant secondary metabolites synthesis and their regulations under biotic and abiotic constraints. J Plant Biol.

[2] Ashrafian H, Bogle RG (2004). Structure, catalytic mechanism, and evolution of the glutathione transferases. J Intensive Care Soc.

[3] Dixon DP, Skipsey M, Edwards R (2010). Roles for glutathione transferases in plant secondary metabolism. Phytochemistry.

[4] Sandermann H (1992). Plant metabolism of xenobiotics. Trends Biochem Sci.

[5] Chi Y, Cheng Y, Vanitha J, Kumar N, Ramamoorthy R, Ramachandran S (2011). Expansion mechanisms and functional divergence of the glutathione S-transferase family in sorghum and other higher plants. Genome Biol.

[6] Dixon DP, Lapthorn A, Edwards R. Plant glutathione transferases. Genome Biol. 2002;3:1–10. 10.1186/gb-2002-3-3-reviews3004. 10.1186/gb-2002-3-3-reviews3004 PMC139027 11897031

[7] Yang G, Xu Z, Peng S, Sun Y, Jia C, Zhai M (2016). In planta characterization of a tau class glutathione S-transferase gene from Juglans regia (JrGSTTau1) involved in chilling tolerance. Plant Cell Rep.

[8] Marrs KA, Alfenito MR, Lloyd AM, Walbot V (1995). A glutathione S-transferase involved in vacuolar transfer encoded by the maize gene Bronze-2. Nature.

[9] Zhao J, Dixon RA (2010). The “ins” and “outs” of flavonoid transport. Trends Plant Sci.

[10] Dixon DP, Cole DJ, Edwards R (1999). Dimerisation of maize glutathione transferases in recombinant bacteria. Plant Mol Biol.

[11] Puglisi I, Lo Cicero L, Lo Piero AR (2013). The glutathione S-transferase gene superfamily: an in silico approach to study the post translational regulation. Biodegradation.

[12] Isoforms I, Sommer A, Bo P (1999). Characterization of recombinant corn glutathione S-transferase isoforms I, II, III, and IV. Pestic Biochem Physiol.

[13] Edwards R, Dixon DP (2005). Plant glutathione transferases. Methods Enzymol.

[14] Mohsenzadeh S, Esmaeili M, Moosavi F, Shahrtash M, Saffari B, Mohabatkar H (2011). Plant glutathione S-transferase classification, structure and evolution. African J Biotechnol.

[15] Kayum MA, Nath UK, Park JI, Biswas MK, Choi EK, Song JY (2018). Genome-wide identification, characterization, and expression profiling of glutathione S-transferase (GST) family in pumpkin reveals likely role in cold-stress tolerance. Genes (Basel).

[16] Nianiou-Obeidat I, Madesis P, Kissoudis C, Voulgari G, Chronopoulou E, Tsaftaris A (2017). Plant glutathione transferase-mediated stress tolerance: functions and biotechnological applications. Plant Cell Rep.

[17] Jain M, Ghanashyam C, Bhattacharjee A (2010). Comprehensive expression analysis suggests overlapping and specific roles of rice glutathione S-transferase genes during development and stress responses. BMC Genomics.

[18] Sappl PG, Carroll AJ, Clifton R, Lister R, Whelan J, Harvey Millar A (2009). The Arabidopsis glutathione transferase gene family displays complex stress regulation and co-silencing multiple genes results in altered metabolic sensitivity to oxidative stress. Plant J.

[19] Lai B, You Y, Zhang L, Wang Q, Chen F, Luo G (2021). Identification and functional characterization of RsGST1, an anthocyanin-related glutathione S-transferase gene in radish. J Plant Physiol.

[20] Zhuge XL, Xu H, Xiu ZJ, Yang HL (2020). Biochemical functions of glutathione S-transferase family of salix babylonica. Front Plant Sci.

[21] Han XM, Yang ZL, Liu YJ, Yang HL, Zeng QY (2018). Genome-wide profiling of expression and biochemical functions of the Medicago glutathione S-transferase gene family. Plant Physiol Biochem.

[22] Zhao YW, Wang CK, Huang XY, Hu DG (2021). Genome-wide analysis of the glutathione s-transferase (Gst) genes and functional identification of mdgstu12 reveals the involvement in the regulation of anthocyanin accumulation in apple. Genes (Basel).

[23] Xu J, Tian YS, Xing XJ, Peng RH, Zhu B, Gao JJ (2016). Over-expression of AtGSTU19 provides tolerance to salt, drought and methyl viologen stresses in Arabidopsis. Physiol Plant.

[24] Dong Y, Li C, Zhang Y, He Q, Daud MK, Chen J (2016). Glutathione s-transferase gene family in Gossypium raimondii and G. arboreum: Comparative genomic study and their expression under salt stress. Front Plant Sci.

[25] Kao CW, Bakshi M, Sherameti I, Dong S, Reichelt M, Oelmüller R (2016). A Chinese cabbage (Brassica campetris subsp. Chinensis) τ-type glutathione-S-transferase stimulates Arabidopsis development and primes against abiotic and biotic stress. Plant Mol Biol.

[26] Chan C, Lam HM (2014). A putative lambda class glutathione S-transferase enhances plant survival under salinity stress. Plant Cell Physiol.

[27] Jia B, Sun M, Sun X, Li R, Wang Z, Wu J (2016). Overexpression of GsGSTU13 and SCMRP in Medicago sativa confers increased salt-alkaline tolerance and methionine content. Physiol Plant.

[28] Xu J, Xing XJ, Tian YS, Peng RH, Xue Y, Zhao W (2015). Transgenic Arabidopsis plants expressing tomato glutathione S-transferase showed enhanced resistance to salt and drought stress. PLoS ONE.

[29] Wang Z, Huang S, Jia C, Liu J, Zhang J, Xu B (2013). Molecular cloning and expression of five glutathione S-transferase (GST) genes from Banana (Musa acuminata L. AAA group, cv. Cavendish). Plant Cell Rep.

[30] Liu D, Liu Y, Rao J, Wang G, Li H, Ge F (2013). Overexpression of the glutathione S-transferase gene from Pyrus pyrifolia fruit improves tolerance to abiotic stress in transgenic tobacco plants. Mol Biol.

[31] Yadav SK (2009). Cold stress tolerance mechanisms in plants. Sustain Agric.

[32] Pennington HG, Gheorghe DM, Damerum A, Pliego C, Spanu PD, Cramer R (2016). Interactions between the powdery mildew effector BEC1054 and barley proteins identify candidate host targets. J Proteome Res.

[33] Wang Q, Guo J, Jin P, Guo M, Guo J, Cheng P (2022). Glutathione S-transferase interactions enhance wheat resistance to powdery mildew but not wheat stripe rust. Plant Physiol.

[34] Kumar S, Asif MH, Chakrabarty D, Tripathi RD, Dubey RS, Trivedi PK (2013). Expression of a rice Lambda class of glutathione S-transferase, OsGSTL2, in Arabidopsis provides tolerance to heavy metal and other abiotic stresses. J Hazard Mater.

[35] Bernard F, Dumez S, Brulle F, Lemière S, Platel A, Nesslany F (2016). Antioxidant defense gene analysis in Brassica oleracea and Trifolium repens exposed to Cd and/or Pb. Environ Sci Pollut Res.

[36] Chronopoulou E, Madesis P, Asimakopoulou B, Platis D, Tsaftaris A, Labrou NE (2012). Catalytic and structural diversity of the fluazifop-inducible glutathione transferases from Phaseolus vulgaris. Planta.

[37] Islam S, Sajib SD, Jui ZS, Arabia S, Islam T, Ghosh A (2019). Genome-wide identification of glutathione S-transferase gene family in pepper, its classification, and expression profiling under different anatomical and environmental conditions. Sci Rep.

[38] Jiang HW, Liu MJ, Chen IC, Huang CH, Chao LY, Hsieh HL (2010). A glutathione s-transferase regulated by light and hormones participates in the modulation of arabidopsis seedling development. Plant Physiol.

[39] Yamaguchi-Shinozaki K, Shinozaki K (2005). Organization of cis-acting regulatory elements in osmotic- and cold-stress-responsive promoters. Trends Plant Sci.

[40] Schaeffer S, Koepke T, Dhingra A (2012). Tobacco: A Model Plant for Understanding the Mechanism of Abiotic Stress Tolerance. Improv Crop Resist to Abiotic Stress.

[41] Zhou J, Li XQ, Huang Y, et al. Comprehensive Evaluation of Tobacco-growing Soil Fertility in Karst Mountain Area—Take Xixiu District of Anshun As an Example. J Mt Agric Biol. 2021;40:29-35.

[42] Lo Cicero L, Madesis P, Tsaftaris A, Lo Piero AR (2015). Tobacco plants over-expressing the sweet orange tau glutathione transferases (CsGSTUs) acquire tolerance to the diphenyl ether herbicide fluorodifen and to salt and drought stresses. Phytochemistry.

[43] Kim YJ, Lee OR, Lee S, Kim KT, Yang DC (2012). Isolation and characterization of a theta glutathione S-transferase gene from Panax ginseng meyer. J Ginseng Res.

[44] Pu T, Mo Z, Su L, Yang J, Wan K, Wang L (2022). Genome-wide identification and expression analysis of the ftsH protein family and its response to abiotic stress in Nicotiana tabacum L. BMC Genomics.

[45] Sun W, Ma Z, Liu M (2020). Cytochrome P450 family: Genome-wide identification provides insights into the rutin synthesis pathway in Tartary buckwheat and the improvement of agricultural product quality. Int J Biol Macromol.

[46] Wani SH, Kumar V, Shriram V, Sah SK (2016). Phytohormones and their metabolic engineering for abiotic stress tolerance in crop plants. Crop J.

[47] Vijayakumar H, Thamilarasan SK, Shanmugam A, Natarajan S, Jung HJ, Park JI (2016). Glutathione transferases superfamily: cold-inducible expression of distinct GST genes in Brassica oleracea. Int J Mol Sci.

[48] Islam S, Rahman IA, Islam T, Ghosh A (2017). Genome-wide identification and expression analysis of glutathione S-transferase gene family in tomato: gaining an insight to their physiological and stress-specific roles. PLoS ONE.

[49] Soranzo N, Sari Gorla M, Mizzi L, De Toma G, Frova C (2004). Organisation and structural evolution of the rice glutathione S-transferase gene family. Mol Genet Genomics.

[50] Islam MS, Choudhury M, Majlish ANK, Islam T, Ghosh A (2018). Comprehensive genome-wide analysis of Glutathione S-transferase gene family in potato (Solanum tuberosum L.) and their expression profiling in various anatomical tissues and perturbation conditions. Gene.

[51] Hao Y, Xu S, Lyu Z, Wang H, Kong L, Sun S (2021). Comparative analysis of the glutathione S-Transferase gene family of four triticeae species and transcriptome analysis of gst genes in common wheat responding to salt stress. Int J Genomics.

[52] Battey JND, Sierro N, Ivanov N V. Characterizing the Genome of Nicotiana tabacum. 2020.

[53] Yu J, Wang J, Lin W, Li S, Li H, Zhou J (2005). The genomes of oryza sativa: a history of duplications. PLoS Biol.

[54] Sato S, Tabata S, Hirakawa H, Asamizu E, Shirasawa K, Isobe S (2012). The tomato genome sequence provides insights into fleshy fruit evolution. Nature.

[55] Hulse-Kemp AM, Maheshwari S, Stoffel K, Hill TA, Jaffe D, Williams SR (2018). Reference quality assembly of the 3.5-Gb genome of Capsicum annuum from a single linked-read library. Hortic Res.

[56] Flagel LE, Jonathan FW (2009). Gene duplication and evolutionary novelty in plants. New Phytol.

[57] Walkowiak S, Gao L, Monat C, Haberer G, Kassa MT, Brinton J (2020). Multiple wheat genomes reveal global variation in modern breeding. Nature.

[58] Wang R, Ma J, Zhang Q, Wu C, Zhao H, Wu Y (2019). Genome-wide identification and expression profiling of glutathione transferase gene family under multiple stresses and hormone treatments in wheat (Triticum aestivum L.). BMC Gen.

[59] Narusaka Y, Nakashima K, Shinwari ZK, Sakuma Y, Furihata T, Abe H (2003). Interaction between two cis-acting elements, ABRE and DRE, in ABA-dependent expression of Arabidopsis rd29A gene in response to dehydration and high-salinity stresses. Plant J.

[60] Jia B, Wang Y, Zhang D, Li W, Cui H, Jin J (2021). Genome-wide identification, characterization and expression analysis of soybean chyr gene family. Int J Mol Sci.

[61] Guo Y, Halfter U, Ishitani M, Zhu JK (2001). Molecular characterization of functional domains in the protein kinase SOS2 that is required for plant salt tolerance. Plant Cell.

[62] Zhu JK (2016). Abiotic stress signaling and responses in plants. Cell.

[63] Chi Y, Cheng Y, Vanitha J, Kumar N, Ramamoorthy R, Ramachandran S (2011). Expansion mechanisms and functional divergence of the glutathione S-transferase family in sorghum and other higher plants. DNA Res.

[64] Xu J, Zheng AQ, Xing XJ, Chen L, Fu XY, Peng RH (2018). Glutathione S transferase gene ( VvGSTF13) Show enhanced tolerance to abiotic stress. Biochem.

[65] Kumar S, Asif MH, Chakrabarty D, Tripathi RD, Dubey RS, Trivedi PK (2013). Differential expression of rice lambda class gst gene family members during plant growth, development, and in response to stress conditions. Plant Mol Biol Report.

[66] Moons A (2005). Regulatory and functional interactions of plant growth regulators and plant glutathione S-transferases (GSTs). Vitam Horm.

[67] Moons A (2003). Osgstu3 and osgtu4, encoding tau class glutathione S-transferases, are heavy metal- and hypoxic stress-induced and differentially salt stress-responsive in rice roots. FEBS Lett.

[68] Guo AY, Zhu QH, Chen X, Luo JC (2007). GSDS: a gene structure display server. Yi Chuan.

[69] Bailey TL, Boden M, Buske FA, Frith M, Grant CE, Clementi L (2009). MEME suite: tools for motif discovery and searching. Nucleic Acids Res.

[70] Chen C, Chen H, Zhang Y, Thomas HR, Frank MH, He Y (2020). TBtools: an integrative toolkit developed for interactive analyses of big biological data. Mol Plant.

[71] Mo Z, Luo W, Pi K, Duan L, Wang P, Ke Y (2022). Comparative transcriptome analysis between inbred lines and hybrids provides molecular insights into K+ content heterosis of tobacco (Nicotiana tabacum L.). Front Plant Sci.

[72] Livak KJ, Schmittgen TD (2001). Analysis of relative gene expression data using real-time quantitative PCR and the 2^-ΔΔCT^ method. Methods.

